# Distinct gene expression signatures in Lynch syndrome and familial colorectal cancer type X

**DOI:** 10.1186/1753-6561-7-S2-P52

**Published:** 2013-04-04

**Authors:** Mev Dominguez Valentin, Cristina Therkildsen, Srinivas Veerla, Mats Jönsson, Inge Bernstein, Mef Nilbert

**Affiliations:** 1 Department of Oncology, Clinical Sciences, Lund University, 22185 Lund, Sweden; 2 Clinical Research Centre, Hvidovre University Hospital, Copenhagen University, 2650 Hvidovre, Denmark; 3 HNPCC Register, Department of Gastroenterology, Hvidovre University Hospital, Copenhagen University, 2650 Hvidovre, Denmark

## Background

Heredity is estimated to cause at least 20% of colorectal cancer. The hereditary nonpolyposis colorectal cancer (HNPCC) subset is divided into Lynch syndrome and familial colorectal cancer type X (FCCTX) based on presence of mismatch repair gene defects. We addressed the gene expression signatures in colorectal cancer linked to Lynch syndrome and FCCTX with the aim to identify diagnostic discriminators and to map signaling pathways relevant to hereditary colorectal carcinogenesis.

## Patients and methods

RNA extracted from 123 colorectal cancers, including 39 Lynch syndrome tumors, 37 FCCTX tumors and 47 sporadic tumors was analyzed using the whole-genome c-DNA-mediated annealing, selection, extension, and ligation (WG-DASL) assay containing 18k genes, where after key targets were validated by real time quantitative RT-PCR (qRT-PCR).

## Results

Colorectal cancers linked to Lynch syndrome and FCCTX showed distinct gene expression profiles, which by significance analysis of microarrays (SAM) differed by 2188 genes. Functional pathways involved were related to G-protein coupled receptor signaling, oxidative phosphorylation, cell cycle function and mitosis and genes herein proved deregulated using qRT-PCR.

## Conclusions

Distinct genetic profiles and deregulation of different canonical pathways apply to Lynch syndrome and FCCTX and key targets herein may be relevant to pursue in relation to refined diagnostic and therapeutic strategies for hereditary colorectal cancer.

## Financial support

Financial support was granted from the Danish and the Swedish Cancer Funds, from the Swedish Research Council, the Lundbeck foundation, the Nilsson Cancer Fund, the Kamprad Cancer Fund and the Hvidovre University Hospital, Denmark.

